# Relapsing Guillain–Barré syndrome associated with Graves’ disease and papillary thyroid carcinoma: a case report and literature review

**DOI:** 10.3389/fimmu.2026.1791151

**Published:** 2026-05-18

**Authors:** Yushu Jiang, Rui Pang, Shuhua Dai, Ting Zhao, Jiewen Zhang, Wei Li

**Affiliations:** 1 Department of Neurology, Henan Joint International Research Laboratory of Accurate Diagnosis, Treatment, Research and Development, Henan Provincial People’s Hospital, People’s Hospital of Zhengzhou University, Zhengzhou, Henan, China; 2 Department of Neurology, Zhoukou Central Hospital, Zhoukou, Henan, China

**Keywords:** Guillain–Barré syndrome, Graves’ disease, papillary thyroid carcinoma, immune dysregulation, case report

## Abstract

Guillain–Barré syndrome (GBS) is an acute immune-mediated peripheral neuropathy typically characterized by a monophasic course; however, atypical or relapsing presentations suggest that sustained immune dysregulation contribute to disease heterogeneity. We report a 33-year-old man with progressive limb weakness diagnosed as motor-predominant acute inflammatory demyelinating polyradiculoneuropathy (AIDP), supported by albuminocytologic dissociation and demyelinating features on nerve conduction studies with preserved sensory conduction. Immunological evaluation revealed B lymphocyte expansion, elevated interleukin-6 and interleukin-8 levels, and strong thyroid autoantibody positivity consistent with Graves’ disease, while anti-ganglioside and paraneoplastic antibodies were negative. The clinical course was marked by immune-triggered relapses and the patient responded well to intravenous immunoglobulin (IVIg) and FcRn inhibition with efgartigimod. Further investigation identified papillary thyroid carcinoma harboring a BRAF V600E mutation, after which neurological symptoms stabilized following thyroidectomy. This case illustrates a rare concurrence of relapsing AIDP, Graves’ disease, and papillary thyroid carcinoma, and supports the hypothesis that persistent systemic immune activation underlies relapsing GBS, rather than a chronic demyelinating process. Clinically, it highlights the importance of considering underlying autoimmune and neoplastic conditions in atypical or relapsing GBS to guide individualized management.

## 1 Introduction

Guillain–Barré syndrome (GBS) is an acute immune-mediated peripheral neuropathy classically characterized by a monophasic disease course and antibody-dependent mechanisms targeting peripheral nerves (1). Although antecedent infections represent the most common triggers, GBS has also been reported in association with systemic autoimmune conditions (2), supporting the concept that broader immune dysregulation may contribute to disease susceptibility and heterogeneity in selected patients.

Autoimmune thyroid diseases, particularly Graves’ disease, have been sporadically reported in association with GBS (3, 4). In these cases, neurological manifestations often develop during active thyrotoxicosis and typically improve following standard immunotherapy combined with restoration of euthyroid status. However, the clinical presentations are heterogeneous, and the immunopathogenic links between thyroid autoimmunity and peripheral nerve involvement remain incompletely understood. In contrast, the coexistence of GBS with thyroid malignancies is exceedingly rare. The few available reports involving papillary thyroid carcinoma (PTC) describe atypical or severe neurological courses, frequently in the absence of classical paraneoplastic antibodies, leaving the underlying mechanisms largely speculative (5, 6). Here, we report a patient with relapsing acute inflammatory demyelinating polyradiculoneuropathy (AIDP) occurring in the concurrent setting of autoimmune hyperthyroidism and occult PTC. This case is notable for its relapsing but non-progressive disease course, detailed immunological characterization, responsiveness to IgG-targeted therapies, and neurological stabilization following tumor resection. By integrating clinical, immunological, and oncological findings, this report expands the clinical spectrum of GBS and provides insights into potential systemic immune dysregulation spanning autoimmune and tumor-associated contexts.

## 2 Case presentation

A graphical summary of the clinical course is provided in **Figure 1**.

**Figure 1:**
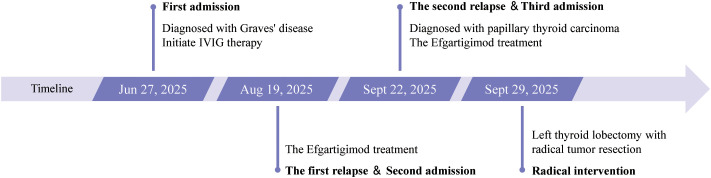
Timeline of clinical course, diagnostic findings, and therapeutic interventions.

A 33-year-old Chinese man was admitted to the Department of Neurology, Henan Provincial People’s Hospital on June 27, 2025, with progressive limb weakness. His main complaints were bilateral upper limb weakness lasting 20 days, which had worsened and was accompanied by fatigue of the left lower limb for 2 days. In early June 2025, the patient noticed a sensation of tightness in both hands upon awakening without an identifiable trigger, followed by gradually progressive weakness in both upper limbs. He experienced difficulty performing fine motor tasks, including grasping objects and twisting bottle caps. Two days prior to admission, new-onset weakness and fatigue developed in the left lower limb. He was evaluated at a local hospital, where electromyography suggested radicular involvement and bilateral upper limb peripheral neuropathy. Neurotrophic treatment with methylcobalamin and B vitamins was administered without clinical improvement. Throughout the disease course, the patient remained fully conscious with preserved mental status. There were no bladder or bowel disturbances. Appetite, sleep, and body weight remained stable. He had no prior history of neurological, autoimmune, or endocrine disorders and no family history of neuromuscular disease or malignancy. He denied recent vaccination or antecedent respiratory or gastrointestinal infection.

On admission, vital signs were stable. Neurological examination revealed an alert and oriented patient with fluent speech and intact higher cortical functions. Cranial nerve examination was unremarkable. Motor examination showed reduced bilateral handgrip strength (Medical Research Council [MRC] grade III^+^) and decreased strength of bilateral forearm flexors (MRC grade IV^−^). Strength of the forearm extensors, proximal upper limbs, and bilateral lower extremities was preserved (MRC grade V). Muscle tone was normal in all extremities. Deep tendon reflexes were absent in the bilateral biceps, reduced in the triceps, and symmetrically preserved in the brachioradialis and lower extremities. Pathological reflexes were absent. Coordination, sensory examination, and meningeal signs were normal.

Routine laboratory investigations, including complete blood count, inflammatory markers (C-reactive protein and procalcitonin), liver and renal function tests, and tumor markers, were within normal limits. Cerebrospinal fluid (CSF) examination revealed an opening pressure of 230 mmH_2_O, a white blood cell count of 2 × 10^6^/L, elevated protein concentration (0.504 g/L), and normal glucose and chloride levels, consistent with albuminocytologic dissociation. Nerve conduction studies demonstrated a multifocal pattern of motor nerve involvement in all four limbs, with predominantly demyelinating features affecting both proximal and distal nerve segments, while sensory nerve conduction velocities were preserved—findings consistent with motor-predominant AIDP (**Supplementary Table 1**). Immunological testing showed negative results for anti-ganglioside antibodies and paraneoplastic antibodies. Notably, the absolute B lymphocyte count (560/μL) and percentage (25.03%) were elevated. Serum cytokine profiling demonstrated increased levels of interleukin-6 (8.24 pg/mL) and interleukin-8 (143.04 pg/mL), along with elevated complement C3 levels (1.51 g/L). Immunofixation electrophoresis revealed no monoclonal immunoglobulin bands.

Thyroid function tests showed markedly suppressed thyroid-stimulating hormone (TSH, 0.01 μIU/mL) with elevated free triiodothyronine (FT3) and total triiodothyronine (TT3), consistent with thyrotoxicosis. Thyroid autoantibody testing revealed markedly elevated thyrotropin receptor antibodies (TRAb, 13.83 IU/L) and anti-thyroid peroxidase antibodies (TPOAb, 711.79 IU/mL), while anti-thyroglobulin antibodies (TgAb) were within the normal range (2.89 IU/mL), supporting a diagnosis of Graves’ disease. Thyroid ultrasonography identified a solid hypoechoic nodule in the left thyroid lobe classified as C-TIRADS 4b. Whole-body ^18^F-FDG PET/CT demonstrated focal increased FDG uptake in a small hypodense nodule of the left thyroid lobe (**Figure 2**). Fine-needle aspiration biopsy performed on September 26, 2025, confirmed PTC (Bethesda category V), with detection of a BRAF V600E mutation (**Figure 3**).

**Figure 2:**
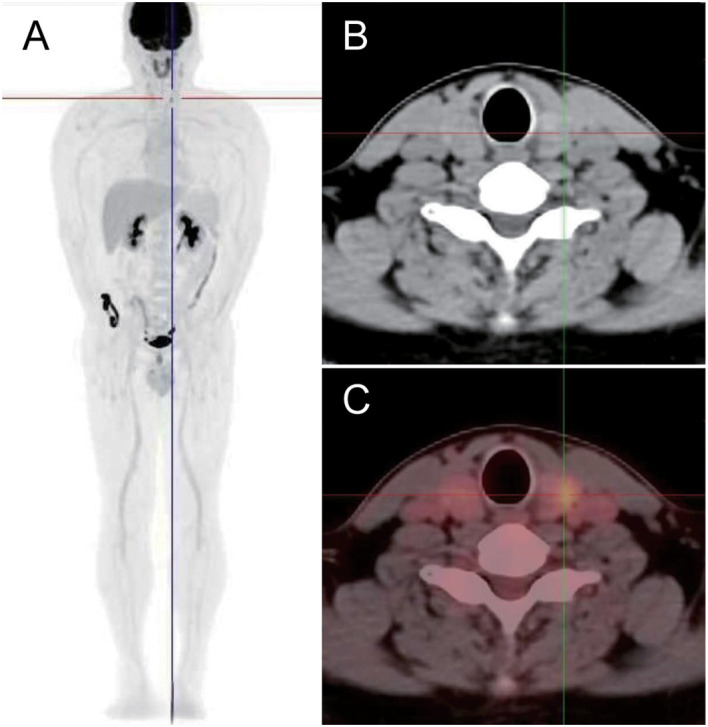
Whole-body ^18^F-FDG PET/CT imaging findings. Whole-body ^18^F-FDG PET/CT demonstrated heterogeneous thyroid density with a hypodense nodule in the left thyroid lobe (approximately 6 × 4 mm), showing increased FDG uptake (SUVmax = 5.61). **(A)** Three-dimensional maximum intensity projection (3D-MIP) image. **(B)** Axial CT image showing the hypodense nodule. **(C)** PET/CT fusion image demonstrating focal increased FDG uptake corresponding to the thyroid lesion.

**Figure 3:**
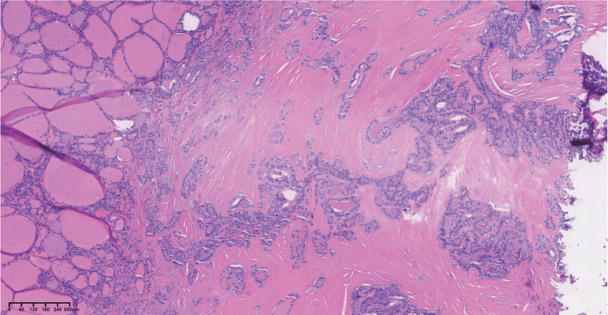
Histopathological features of the thyroid lesion. Low-power photomicrograph (H&E, ×40) showing a thyroid nodule containing tumor cells arranged in irregular glands, cords, and nests, with stromal fibrosis and infiltration into adjacent thyroid tissue. Residual thyroid follicles of variable size are present around the lesion.

Methimazole therapy was initiated after admission to control thyrotoxicosis. Based on the clinical presentation, CSF findings, and electrophysiological features, the patient was diagnosed with motor-predominant AIDP. IVIg was administered at 0.4 g/kg/day for 5 consecutive days starting on July 4, 2025. The patient showed marked clinical improvement and was discharged after recovery of handgrip strength. Following a relapse associated with gingivitis, the patient responded well to FcRn inhibition with efgartigimod. After a second relapse, left thyroid lobectomy with radical tumor resection was performed on September 29, 2025. Neurological symptoms stabilized following surgery.

At 3-month follow-up, the patient showed sustained clinical improvement, with full recovery of muscle strength (MRC grade V). Thyroid function normalized, with TSH 2.24 μIU/mL, FT3 2.77 pmol/L, and TT3 0.76 μg/dL. Thyroid autoantibody levels decreased but remained positive for TRAb (3.63 IU/L) and TPOAb (283.2 IU/mL), whereas TgAb remained within normal limits (1.16 IU/mL). Serum calcium remained within the normal range (2.38 mmol/L).

## 3 Literature review

Previous reports of GBS occurring in association with thyroid disorders are rare and largely limited to isolated case descriptions. A narrative review of the literature identified a small number of reported cases, comprising patients with hyperthyroidism—most commonly autoimmune hyperthyroidism—and a very limited number of cases involving PTC.

Among hyperthyroidism-associated cases, neurological manifestations typically developed during active thyrotoxic states or shortly thereafter. The majority of patients presented with AIDP, although atypical variants such as Miller–Fisher syndrome or pharyngeal–cervical–brachial weakness have been reported. CSF demonstrated albuminocytologic dissociation, and most patients showed favorable neurological outcomes following standard immunotherapy combined with antithyroid treatment.

In contrast, the coexistence of GBS and thyroid malignancy, particularly PTC, is exceedingly rare. The few reported cases have been characterized by severe or atypical neurological courses, including rapid progression, respiratory failure, cranial nerve involvement, or prolonged recovery. Classical paraneoplastic neuronal antibodies were consistently absent, and no definitive infectious triggers were identified, suggesting a non-classical tumor-associated immune mechanism.

Collectively, the available literature underscores the heterogeneity of GBS associated with thyroid disorders and highlights a substantial gap in mechanistic understanding. While hyperthyroidism-associated GBS generally follows a relatively benign and self-limited course, PTC-associated cases appear to represent a distinct and more complex subset. A summary of previously reported cases is provided in **Table 1**.

**Table 1:** Summary of reported cases of GBS associated with thyroid disorders and PTC.

Country	Publication date	Number of cases	Sex	Age(years)	Thyroid disorder	PTC	Immunology findings	GBS variant	Electrophysiological findings	Treatment	Thyroid surgery	Neurological outcome
USA (19)	1964	4	Both females	Mean Age- 36	Graves’ disease/non- toxic goiter	No	NR	NR	NR	Supportive care	Thyroid surgery was performed in 3/4 cases: Case 2 & 3 & 4: Subtotal thyroidectomy. Case 1: No surgery.	Case 1: Complete recovery. Case 2: Partial recovery. Case 3: Poor outcome. Case 4: Significant improvement
Switzerland (20)	2008	1	Female	42	Graves’ disease	No	TSH-receptor Ab (+), Microsomal Ab (+), anti-GQ1b Ab (+)	MFS	Normal	Prednisolone	No	Complete recovery
Belgium (6)	2019	1	Male	35	No	Yes	No	AIDP	Absent motor and sensory action potentials	IVIG, TPE	Total thyroidectomy with left cervical lymph node dissection	Complete recovery
India (11)	2019	1	Female	35	Graves’ disease	No	TSH-receptor Ab (+), Anti-TPO Ab (+)	PCB	Normal	Supportive care	No	Complete recovery
Turkey (21)	2022	1	Female	34	Thyrotoxicosis	No	Anti-GD1a (+), GQ1b (+), and GT1a (+)	BBE/GBS	Myopathic findings with normal nerve conduction studies in the early period.	TPE	No	Complete recovery
Japan (3)	2022	1	Female	55	Graves’ disease	No	Anti-Tg Ab (+), Anti-TPO Ab (+), TSH-receptor Ab (+), anti-GM1 Ab (+)	AIDP	Normal	IVIG	No	Complete recovery
USA (5)	2023	1	Male	75	No	Yes	NR	NR	Not reported	IVIG	No	Poor outcome
Vietnam (4)	2024	1	Male	50	Thyroid storm	No	NR	AIDP	Sensory and motor axonal polyneuropathy	IVIG, TPE	No	Significant improvement
China (our case)	2025	1	Male	33	Graves’ Disease	Yes (BRAF V600E)	TSH-receptor Ab (+), Anti-TPO Ab (+)	AIDP	Motor-predominant demyelinating neuropathy	IVIG, FcRn inhibitor	Yes	Complete recovery

GBS, Guillain–Barré syndrome; AIDP, acute inflammatory demyelinating polyradiculoneuropathy; MFS, Miller Fisher syndrome; PCB, pharyngeal–cervical–brachial variant; BBE, Bickerstaff’s brainstem encephalitis; IVIG, intravenous immunoglobulin; TPE, therapeutic plasma exchange; PTC, papillary thyroid carcinoma; NR, not reported.

## 4 Discussion

### 4.1 Diagnostic considerations

The diagnosis of GBS in this case followed the 2023 EAN/PNS guideline (7). The patient presented with acute-onset symmetric limb weakness and reduced or absent deep tendon reflexes. CSF analysis showed albuminocytologic dissociation, with elevated protein (0.504 g/L) and a normal white blood cell count (2 × 10^6^/L). Nerve conduction studies demonstrated predominantly motor demyelinating features with preserved sensory conduction. These findings are consistent with motor-predominant AIDP.

Differential diagnoses included: (1) Chronic inflammatory demyelinating polyradiculoneuropathy (CIDP) – CIDP was considered unlikely based on disease course and treatment response; (2) Paraneoplastic neuropathy – although papillary thyroid carcinoma was present, all paraneoplastic antibodies were negative, and the neurological course did not follow the subacute progressive pattern typical of paraneoplastic disorders; (3) Other acute polyneuropathies (e.g., porphyria, vasculitic neuropathy, Lyme disease) – excluded by negative laboratory tests and lack of systemic involvement.

Notably, serum anti-ganglioside antibodies were negative. Antibody-negative GBS is not uncommon, particularly in demyelinating subtypes such as AIDP (8). The absence of detectable antibodies does not indicate a non-classical GBS subtype. Instead, it reflects the heterogeneity of immune targets in GBS, where humoral responses may involve unidentified antigens or be complemented by cellular immune mechanisms (9). The clinical response to IVIg and FcRn inhibition further supports the involvement of humoral immune pathways.

### 4.2 Comparison with existing literature

The existing literature indicates that GBS associated with thyroid disorders is rare and heterogeneous (10). In cases associated with autoimmune hyperthyroidism, neurological symptoms often improve after immunotherapy and restoration of euthyroid status (4, 11). In contrast, cases associated with PTC are extremely uncommon and less well characterized. Reported PTC-associated cases have been characterized by atypical severity, prolonged or complicated courses, and an absence of classical paraneoplastic antibodies. These observations suggest that mechanisms beyond post-infectious or canonical paraneoplastic pathways may be involved (5, 6).

The present case adds several novel features. The patient exhibited a relapsing but non-progressive AIDP phenotype in the setting of concomitant autoimmune hyperthyroidism and occult PTC. The disease course was notable for immune-triggered relapses, evidence of B-cell activation with cytokine elevation, a marked clinical response to IgG-targeted therapies, and subsequent neurological stabilization following tumor resection. These features bridge clinical characteristics described in autoimmune and malignancy-associated GBS. They support the hypothesis that sustained systemic immune dysregulation may underlie relapsing GBS in selected patients.

### 4.3 Clinical implications of relapsing AIDP

Although AIDP was diagnosed, the relapsing course in this patient has important clinical implications. Recurrent deterioration after initial improvement raises the differential consideration of treatment-related fluctuations (TRF), recurrent GBS, or acute-onset CIDP.

In this case, several features support a relapsing AIDP phenotype rather than CIDP. The initial progression reached nadir within three weeks. There was no continuous worsening beyond eight weeks. Each relapse occurred after a period of clinical stabilization. Relapses were temporally associated with potential immune triggers. The patient responded rapidly and substantially to short-term immunotherapy intervention. Maintenance therapy was not required.

This pattern is more consistent with TRF or relapsing AIDP than with CIDP (12, 13). Recognizing this pattern is clinically important, as it may influence treatment strategy and prompt evaluation for persistent systemic immune activation.

### 4.4 B cell–driven immune activation

Accumulating evidence suggests that humoral immune mechanisms play an important role in both GBS and autoimmune thyroid disease (14, 15). In this case, B lymphocyte expansion, elevated cytokines (IL-6 and IL-8) were observed. Thyroid autoantibodies were also strongly positive. These findings suggest a state of heightened B cell–mediated immune activation.

The patient responed well to IgG-targeted therapies, including IVIg and FcRn inhibition with efgartigimod. This supports the involvement of antibody-dependent immune mechanisms (9). Although no specific pathogenic antibody was identified, the therapeutic efficacy of IgG modulation suggests that circulating autoantibodies or immune complexes may have contributed to disease activity.

Additional mechanisms may also be involved. Both thyroid follicular cells and peripheral nerve membranes are enriched in gangliosides, raising the possibility of cross-reactive immune responses (16). Shared or sequential infectious exposures may contribute to immune activation (14, 17). Together, these factors suggest an immune environment in which Graves’ disease may lower the threshold for GBS onset or relapse.

### 4.5 Potential contribution of PTC to systemic neuroimmune dysregulation

PTC is generally regarded as an indolent tumor. However, increasing evidence indicates that it can influence systemic immune homeostasis (18). In previously reported cases, GBS associated with PTC often showed severe or atypical features, and classical paraneoplastic antibodies were consistently absent (5, 6). These findings suggest that tumor-associated immune effects may occur through indirect mechanisms rather than through established paraneoplastic pathways.

In this case, a BRAF V600E–mutated PTC was identified. This raises the possibility of sustained immune stimulation driven by tumor-related inflammatory signaling. However, this observation remains speculative and cannot establish a direct mechanistic link. Neurological symptoms stabilized following thyroidectomy. This temporal association provides indirect clinical support for a contributory role of the tumor. Thyroid autoantibody levels also decreased in parallel with neurological recovery. These findings support a potential role of systemic immune modulation.

Partial thyroidectomy may have reduced the burden of autoimmune-active thyroid tissue. This could have contributed to decreased systemic immune activation. However, this remains speculative and cannot be distinguished from other concurrent therapeutic effects. Overall, PTC may have acted as a chronic immune-modifying factor. It may have amplified pre-existing autoimmune activity and lowered the threshold for GBS relapse.

### 4.6 Limitations

This study has several limitations. First, this is a single case report and cannot establish a causal or mechanistic relationship between PTC and GBS relapse. Second, although immunological abnormalities were observed, no specific pathogenic antibody was identified. The underlying mechanisms therefore remain speculative.

## 5 Conclusion

This case describes a rare concurrence of relapsing AIDP, Graves’ disease, and papillary thyroid carcinoma. The relapsing but non-progressive disease course, evidence of B cell–mediated immune activation, and a clinical response to IgG-targeted therapies, supports a role for sustained systemic immune dysregulation.

Clinically, this case highlights the importance of evaluating underlying autoimmune and neoplastic conditions in patients with recurrent or atypical GBS. Although causality cannot be established, the temporal association between tumor resection and neurological stabilization suggests a potential interaction between tumor-related immune modulation and peripheral nerve autoimmunity.

## 6 Patient perspective

“At first, I thought the weakness in my hands was just fatigue. When the symptoms kept coming back, I became anxious and confused, especially when treatments that initially worked did not last. Learning that I had a thyroid disease and later a thyroid tumor was unexpected and frightening. After receiving different treatments and eventually undergoing surgery, my condition finally became stable. I am grateful that the doctors continued searching for the underlying cause instead of only treating the symptoms.”

## Data Availability

The original contributions presented in the study are included in the article/**Supplementary Material**. Further inquiries can be directed to the corresponding author.
