# Supplementary material for: Relapsing Guillain–Barré syndrome associated with Graves’ disease and papillary thyroid carcinoma: a case report and literature review
Source: Front Immunol. 2026 May 18;17:1791151. doi: 10.3389/fimmu.2026.1791151 (PMC13222997; doi:10.3389/fimmu.2026.1791151)
Supplement: Supplementary file 1 [file Table1.docx]

**Supplementary table 1. Summary of nerve conduction study findings.**

| **Nerve / Parameter** | **Side** | **Latency** | **Amplitude** | **Conduction Velocity** | **F-wave** |
| --- | --- | --- | --- | --- | --- |
| Median nerve (motor) | Bilateral | Prolonged | Normal | Slowed | Delayed |
| Ulnar nerve (motor) | Bilateral | Prolonged | Normal | Slowed | Delayed |
| Posterior tibial nerve (motor) | Bilateral | Prolonged | Normal | Slowed | Delayed |
| Common peroneal nerve (motor) | Bilateral | Prolonged | Normal | Slowed | Normal |
| Median nerve (sensory) | Bilateral | Normal | Normal | Normal | — |
| Ulnar nerve (sensory) | Bilateral | Normal | Normal | Normal | — |
| Superficial peroneal nerve (sensory) | Bilateral | Normal | Normal | Normal | — |
